# Targeting cancer stem cells for reversing therapy resistance: mechanism, signaling, and prospective agents

**DOI:** 10.1038/s41392-020-00430-1

**Published:** 2021-02-15

**Authors:** He-Ming Zhou, Ji-Gang Zhang, Xue Zhang, Qin Li

**Affiliations:** Department of Clinical Pharmacy, Shanghai General Hospital, Shanghai Jiao Tong University School of medicine, No.100 Haining Road, 200080 Shanghai, People’s Republic of China

**Keywords:** Cancer stem cells, Cancer therapy

## Abstract

Cancer stem cells (CSCs) show a self-renewal capacity and differentiation potential that contribute to tumor progression and therapy resistance. However, the underlying processes are still unclear. Elucidation of the key hallmarks and resistance mechanisms of CSCs may help improve patient outcomes and reduce relapse by altering therapeutic regimens. Here, we reviewed the identification of CSCs, the intrinsic and extrinsic mechanisms of therapy resistance in CSCs, the signaling pathways of CSCs that mediate treatment failure, and potential CSC-targeting agents in various tumors from the clinical perspective. Targeting the mechanisms and pathways described here might contribute to further drug discovery and therapy.

## Introduction

**Fig. 1:**
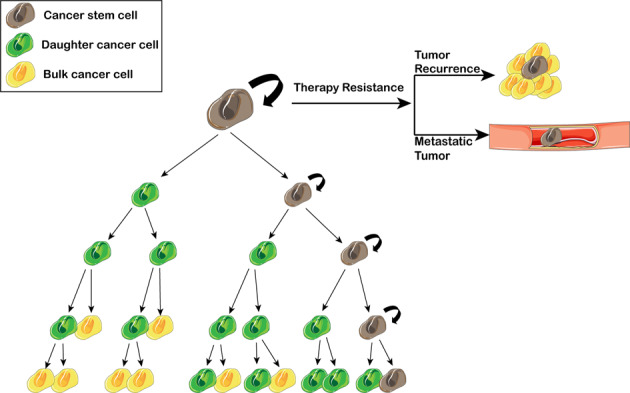
Poor response to therapy due to CSCs. In heterogeneous tumors that contain CSCs, though non-CSCs are ablated, CSC will sustain tumor growth for its ability of self-renewing, then no long-term tumor recurrence or metastatic tumor will be observed

Therapy resistance is becoming a major problem in medicine; while patients initially respond to treatment, sustained administration frequently results in therapy resistance along with a poor prognosis. Both genetic and nongenetic mechanisms enable cancer cells to resist treatment.^1^ Traditionally, cancer is viewed as a homogenous mass of rapidly proliferating cells. Over the last decades, a more complex model, in which cancer tissue is composed of heterogeneous cell populations with a hierarchical organization has replaced the previous one-dimensional view. Cancer stem cells (CSCs) are on top of this hierarchical structure.^2^ Eppert and colleagues^3^ published their pioneering work showing that a defined subset of leukemia cells (CD38+CD34-) was solely responsible for propagating acute myeloid leukemia (AML). A decade later, Al-Hajj’s team showed that as few as 100 cells with a CD44^+^CD24^-^ phenotype could form tumors in mice, whereas tens of thousands of cells with alternate phenotypes failed to form tumors.^4^ Many other teams have also found that only a few fractions of cancer cells can reform secondary tumors after transplantation into immunodeficient mice. This cluster of cells are commonly described using special terms such as CSCs, tumor propagating cells, tumor progenitor cells (TPCs) and cancer-initiating cells (CICs). In many adult tissues, stem cells (SCs) are responsible for tissue homeostasis and regeneration, and they can give rise to transit-amplifying (TA) cell populations.^5^ Similar to normal tissue SCs, CSCs show self-renewal and are defined by their ability to (i) generate a xenograft that histologically resembles the parent tumor from which it was derived, (ii) be serially transplanted in a xenograft assay thereby showing self-renewal (regenerate), and (iii) generate daughter cells that possess some proliferative capabilities but are unable to initiate or maintain the cancer because they lack intrinsic regenerative potential^6^ (Fig. 1).

The mechanisms of therapy resistance in cancer can be categorized as intrinsic and acquired. Intrinsic mechanisms are due to preexisting factors of the cancer that are present prior to any treatment, thus rendering certain treatments useless. Acquired drug resistance develops during treatment. Accumulating evidence has shown that the expression of markers related to stemness is crucial for tumor maintenance and that these molecule also mediate resistance. In most cases, tumor recurrence is the result of a resistant CSC (intrinsic or acquired) in the primary tumor and its sphere formation^7^ and self-renewing abilities^8^ (Fig. 1). Based on the “seed and soil” theory, at a distant site, a resistant CSC can drive metastasis and then form a metastatic tumor^9^ (Fig. 1); thus, an increase in the CSC signature in tumors is associated with a worse prognosis.^3^

Based on the functional CSC concept, CSCs are naturally resistant to chemo- or radiotherapy, indicating they can survive after chemoradiotherapy and develop into a new cancer. However, understanding the properties of CSCs is the first step. The eventual goal is to investigate why CSCs can escape treatment, be retained, and form a new carcinoma.

### Identification of CSCs

The classical definition of CSCs is a rare subpopulation of cells endowed with the capacity for self-renew and tumor-generating potential. Therefore, different methods have been developed and are currently exploited to isolate CSCs from patient-derived tumors or cancer cell lines in vitro. In this context, specific patterns of biomarkers that identify CSCs have been determined for some solid tumors such as CD44^+^CD24^-^ for breast cancer (Table 1). However, increasing findings have showed that the previously defined CSC population is still heterogeneous, and thus, researchers must further enrich these cells by additional differentially expressed markers.

**Table 1:** Identification of cancer stem cells in human cancer

Classification	Markers	Function and role
Isolation markers	CD133	A common CSC marker in various cancers. CD133^+^Nestin^+^ is better for CSCs in glioma.^15,16^ CD133^+^CD44^+^ is better for CSCs in HCC19 and colon tumor.^18^ CD133^+^ALDH^+^^19,20^/ CD133^+^L1CAM^+^^21^ is better for CSCs in OC.
	CD44	A common CSC marker in various cancers. CD44^+^CD133^+^^28^/CD44^+^EpCAM^+^^23^/CD44^+^ALDH^+^^19,29^ is better for CSCs in CRC. CD44^+^c-Met^+^^30^ is better for CSCs in pancreatic cancer; CD44^+^ALDH^+^^32^/ CD44v8-10^34^ is better for CSCs in GC.
	EpCAM	A common CSC marker in various cancers. EpCAM^+^CD44^+^ CD166^+^^23^ is better for CSCs in CRC. EpCAM^+^ CD133^+^^9^ is better for CSCs in HCC
	ALDH	An enzyme that is identified as a common CSC marker in various cancers. ALDH^high^ CD44^+^CD24^-^/ALDH^high^ CD133^+^CD44^+^^41^ is better for CSCs in breast cancer. ALDH^+^CD133^+^^42^ is better for CSCs in HCC.
	CD90	A common CSC marker in various cancers. CD90^+^CD44^+^^49^ is better for CSCs in lung cancer cell.
Intracellular markers	Oct4	Oct4 is a homeodomain transcription factor by binding to octamers, and regulates the expression of many genes.^52^
	Nanog	Nanog is a homeobox transcription factor, And plays a crucial role in the second embryonic cell-fate specification.^62^
	Sox2	Sox2 has an important function in the early development and maintenance of undifferentiated ESCs.
SP fraction	Hoechst 33342- negative population	SP cells can be separated by fluorescence screening after the outflow of Hoechst 33342. And SP cells have high homology, self-renewal and multidirectional differentiation potential.^74^
Noncoding RNAs	Circ008913	Regulate CSC phenotype in nasopharyngeal carcinoma cell line.^91^
	CirGprc5a	Regulate CSC in bladder tumor.^92^
	Circ001680	Promote CSC in CRC and induce irinotecan resistance.^93^
	CircLgr4	Regulate CSC in CRC.^94^
	LncTCF7	Promote CSC by Wnt signaling pathway.^95^
	Lnc-β-Catm	Promote CSC together with Wnt.^96^
	H19	Regulate CSC in breast cancer^97^ and HCC.^98^

#### Isolation markers

CSCs can be isolated through different biomarkers on the cell surface by fluorescence-activated cell sorting (FACS) and magnetic-activated cell sorting (MACS). Classical surface markers, such as CD133, CD44, epithelial cell adhesion molecule (EpCAM) and CD90 are extensively applied. However, some CSC surface markers are shared with normal stem cells. Therefore, multiple markers must be utilized for the accurate targeting of CSCs.

##### CD133

CD133 is a membrane-bound pentaspan glycoprotein first identified in neuroepithelial SCs in mice and later found in human tissues.^10^ CD133 was used as a CSC marker in a series of tumors. In 2004, Singh and colleagues^11^ identified CD133 as a CSC marker in brain TICs because injection of as few as 100 CD133^+^ cells produced a tumor that could be serially transplanted and was a phenocopy of the patient’s original tumor, whereas injection of 10^5^ CD133^−^ cells engrafted but did not result in a tumor. Then, CD133 was identified as a CSC marker in hepatocellular carcinoma (HCC),^12^ glioblastoma,^13^ colon tumors^7^ and ovarian cancers (OCs).^14^ However, CD133 alone cannot always indicate the CSC phenotype. Researchers have thus focused on investigating combined signatures. CD133 combined with Nestin may be an optimal CSC-specific marker in glioma patients.^15,16^ The combination of CD133 and CD44 was used to define a novel HCC subpopulation. CD133^+^CD44^high^ xenografts, but not CD133^+^CD44^−/low^, CD133^−^CD44^high^ or CD133^−^CD44^−/low^ xenografts, produced intrahepatic or lung metastasis in nude mice.^17^ Similarly, Naotsugu Haraguchi’s team showed that the CD133^+^CD44^+^ population may identify TICs in human colon cancer.^18^ In 2009, aldehyde dehydrogenase (ALDH) was reported to contribute to the stemness of the CD133^+^CD44^+^ fraction in colon tumors.^19^ Using ALDH in combination with CD133 to analyze OC cell lines, Ines A Silva observed greater growth in ALDH^+^CD133^+^ cells than ALDH^+^CD133^−^ cells, suggesting a further enrichment of ovarian CSCs in ALDH^+^CD133^+^ cells.^20^ More recently, research showed that double-positive L1 cell adhesion molecule (L1CAM)^+^CD133^+^ cells displayed higher spherogenic and clonogenic properties than L1CAM^−^CD133^−^ cells in OC and indicated radiotherapy resistance.^21^

##### CD44

CD44, a nonkinase transmembrane glycoprotein, is thought to play a role in CSCs.^22^ As mentioned previously, CD44 was first used as a CSC marker in breast cancer.^4^ Then CD44 was identified as a CSC marker in colorectal cancer (CRC),^23,24^ pancreatic cancer,^25^ OC,^26^ gastric cancer,^27^ and others. In CRC, a recent meta-analysis suggested that the combination of CD44 and CD133 indicated an approximately sevenfold increase in the tumorigenic potential, while CD133 alone indicated 1.45-foldchange, and CD44 alone indicated twofold increase.^28^ Moreover, the ability to engraft in vivo in immunodeficient mice was restricted to a minority subpopulation of EpCAM^high^/CD44^+^ epithelial cells in CRC;^23^ furthermore, ALDH combined with either CD44 or CD133 could increase the tumor-initiating ability.^19,29^

Chenwei Li’s team^30^ showed that cells that expressed CD44 (0.5–5%) and c-Met showed a capacity for self-renewal and had the highest tumorigenic potential of all cell populations studied in pancreatic cancer.

In gastric CSCs, CD44^+^ gastric cancer cells showed self-renewal and the ability to form differentiated progeny and gave rise to CD44^−^ cells.^31^ Phu Hung Nguyen showed that CD44 and ALDH are the most specific biomarkers to detect and isolate tumorigenic and chemoresistant gastric CSCs in non-cardia gastric carcinomas independent of the histologic classification of the tumor.^32^ CD44 is a cell surface transmembrane glycoprotein encoded by the CD44 gene, a 20-exon DNA segment,^33^ of which exons 1–5 and 6–20 are spliced together to form CD44s, the standard isoform. In addition, variant exons 6–15 can be alternatively spliced and assembled in different combinations with the standard exons to generate other variant (CD44v) isoforms. From this perspective, further research on gastric cancer found that CD44v8-10 but not CD44s increased the frequency of tumor initiation,^34^ which suggests a strategy to target CSCs in gastric cancer.

##### EpCAM

EpCAM is a transmembrane glycoprotein expressed on the surface of healthy epithelial cells.^35^ EpCAM is increasingly recognized as a specific CSC marker for various tumors such as breast cancer,^4^ colon cancer,^36^ HCC^37^ and pancreatic cancer.^38^ CRC originating from EpCAM^high^/CD44^+^ cells maintained a differentiated phenotype and reproduced the full morphologic and phenotypic heterogeneity of their parental lesions; moreover, CD166 could be an additional differentially expressed marker, for CSC isolation in CRC.^23^ Compared with EpCAM^−^/CD133^−^HCC cells, EpCAM^+^/CD133^+^ cells appear to be a CSC subpopulation in HCC.^9^

##### ALDH

ALDH detoxifies intracellular aldehydes through oxidation and may have a role in the differentiation of SCs through the oxidation of retinoic acid.^39^ In head and neck squamous cell carcinoma (HNSC), ALDH can be used as a single marker of CSCs.^40^ More often, ALDH is combined with other CSC markers: ALDH^high^CD44^+^CD24^-^ and ALDH^high^CD44^+^CD133^+^ cells may be important mediators of breast CSCs.^41^ Further research was conducted and the results revealed the existence of a hierarchical organization in HCC cells with tumorigenic potential as follows: CD133^+^ALDH^+^ > CD133^+^ALDH^−^ > CD133^−^ALDH^−^.^42^

##### CD90

CD90, a glycoprotein, also known as thymocyte differentiation antigen-1 (Thy-1), is a cell adhesion molecule and the smallest member of the immunoglobulin superfamily.^43^ CD90^+^ cells were found to be CSCs in HCC,^44^ as well as prostate cancer,^45^ insulinomas,^46^ OC,^47^ and could predict the response to sorafenib in patients.^48^ Co-expression with the additional surface marker, CD44, produced an even more aggressive phenotype, including a higher metastatic and self-renewal capacity, than that of the CD90^+^CD44^−^ counterparts.^49^

### Intracellular markers

The most important properties of CSCs are self-renewal and the ability to differentiate into one or two more specialized cell types.^50^ Oct4, Nanog and SOX2 are transcription factors that play essential roles during early embryonic development.^50^ On this basis, Shinya Yamanaka’s group found that several transcription factors (Oct4, Sox2, Klf4 and c-Myc) can convert a differentiated cell back to a pluripotent phenotype over the course of a few weeks, thus reprogramming the cells into induced pluripotent stem (iPS) cells.^51^ Upon expression of the reprogramming factors, some cells start to rapidly divide and quickly lose their differentiated cell characteristics with robust downregulation of somatic genes. These transcription factors can be re-expressed or reactivated in CSCs with the ability to self-renew and differentiate.^50^ In theory, the ideal CSC markers are those that are required to maintain their stemness features. Therefore, Oct4, Sox2 and Nanog can be considered as CSC markers.

#### Oct4

Oct4 functions as a homeodomain transcription factor by binding to octamers,^52^ which regulate the expression of many genes. Oct4 was expressed early in the preimplantation embryo and thus regulated early events of murine development.^53,54^ Oct4 was first shown to be associated with cancer by M Monk in 2001.^55^ In 2005, based on CSCs in breast cancer, Ponti and colleagues^56^ found that the CD44^+^CD24^-^ fraction expressed Oct4 and gave rise to new tumors. Oct4^high^ cells have more SC-like traits, such as self-renewal, chemoresistance and xenograft tumorigenicity, than Oct4^low^ cells.^57^ To date, Oct4 has been used to isolate CSCs by Oct4 promoter-mediated activity in breast cancer,^58^ non-small cell lung cancer (NSCLC),^59^ gastric cancer^60^ and HCC.^61^

#### Nanog

Nanog, a homeobox transcription factor, plays a crucial role in the second embryonic cell-fate specification.^62^ This molecule is required for the maintenance of pluripotency but absent from differentiated cells.^63,64^ In 2004, Kristian Almstrup and colleagues^65^ revealed embryonic SC-like features of testicular carcinoma in situ by genome-wide expression profiling: Nanog was upregulated during progression to embryonic carcinoma. In 2010, the expression of Nanog was directly correlated with CSCs (CD133^high^/CD44^high^),^66^ and Nanog^+^ NSCLC cells were shown to exihibit CSC properties.^58^ Furthermore, the status of Nanog determines the switch between cancer cells and CSCs.^67^ Moreover, Nanog expression was associated with enhanced ALDH activity and cellular radioresistance^68^ and chemoreistance.^69^

#### Sox2

Sox2 belongs to the family of high-mobility group transcription factors and has an important function in the early development and maintenance of undifferentiated ESCs. Sox2 is commonly used as a stemness-associated marker in CSC research. Increased expression of Sox2 was observed in CD133^+^ NSCLC cells^70^ and ALDH^high^ cells.^71^ Zhu and colleagues^72^ showed that Sox2 is a marker for CSCs in bladder cancer. In a reporter system (SORE6), which allows the monitoring of viable cells expressing Sox2 and/or Oct4, SORE6^+^ cells were found to be significantly more tumorigenic than SORE6^-^ cells.^60,73^

### Side population (SP) cells

SP cells were discovered in 1996 by M A Goodell in hematopoietic stem cells (HSCs): SP cells were not stained by Hoechst 33342.^74^ SP fractions were shown to protect recipients from lethal irradiation at low cell doses, and to contribute to both lymphoid and myeloid lineages.^74^ SP cells exhibit a low Hoechst 33342 staining pattern because of the high expression of ATP-binding cassette transporters (ABC transporters), especially ABCG2.^75^ Therefore, the ABCG2 transporter is an efficient Hoechst 33342 efflux pump. Moreover, ABCG2 is preferentially expressed by immature human hematopoietic progenitors.^76^ Transplantable HSCs in human fetal liver have an SP phenotype.^77^ Therefore, the SP fraction might indicate a stemness phenotype. Research on AML showed that SP identifies a CD34^+^CD38^-^ progenitor cells.^78^ Then, the SP fraction was used to identify CSCs in solid tumors: Lubna Patrswala’s team first identified the SP fraction in human cancer cells and SP cells were shown to possess some intrinsic SC properties.^79^

Another more recent study showed that P-gp pump function was required for amplification of both phenotypically defined SP cells and functionally defined repopulating cells.^80^ In 2006, SP cells were detected in HCC cells, and the SP fraction presented a CSC phenotype.^81^ Then, the SP fraction was identified in a human nasopharyngeal carcinoma cell line,^82^ OC,^83^ brain tumor,^84^ lung cancer,^85^ especially for CSCs with unknown cell surface markers. Currently, SP analysis is increasingly applied in CSCs research as an indication of stemness^86–88^ and therapy resistance.^89,90^

### Noncoding RNAs

In recent years, research on noncoding RNAs has become increasingly prominent. Many studies have indicated noncoding RNA can be a CSC marker. Circ008913 was reported to be involved in CSC-like properties.^91^ CircGprc5a regulated CSCs in bladder tumors.^92^ Circ001680 could enhance the CSC population in CRC and induce therapeutic resistance to irinotecan.^93^ Moreover, CircLgr4 knockdown inhibited colorectal CSC self-renewal, while CircLgr4 overexpression had the opposite effects.^94^ In addition, several long noncoding RNA (lncRNA)-based regulatory circuits that promote CSCs formation highlight the importance of lncRNAs in CSCs: lncTCF7 promotes CSCs through activation of Wnt signaling,^95^ lnc-β-Catm together with Wnt is required for self-renewal of CSCs,^96^ H19 is associated with CSCs in breast cancer^97^ as well as HCC,^98^ and H19 facilitates angiogenesis by an exosome-mediated mechanism in CSC-like cells.^98^

Overall, although surface markers, SC-specific transcription factors, the SP fraction and noncoding RNAs can be used as CSC markers, the identification and isolation of CSCs in clinical specimens is challenging. The extent to which these marker-identified populations are actual CSCs remains unclear. Moreover, CSC-defining molecules have functions in addition to the roles as markers. Accumulating evidence has shown that these molecules could have specific biological functions in tumor initiation and progression. CD44 is preferentially involved in invasion, adhesion and metastasis, and CD133 tends to be involved in the maintenance of the CSC population. Therefore, instead of solely examing the marker expression in CSCs, we hope to specifically clarify the biological roles played by these markers and the regulatory mechanisms through novel technologies such as live-cell RNA detection and single-cell DNA and RNA sequencing methods.

### Resistance mechanisms of CSCs

**Fig. 2:**
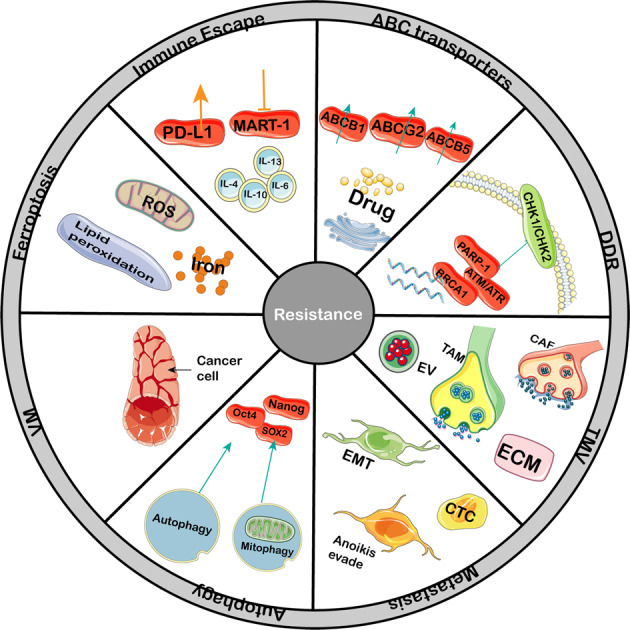
Mechanisms mediating the resistance of CSCs to scores of therapy. Multiple intrinsic and extrinsic resistant mechanisms controlling CSC respond to traditional or novel therapy. In the process or after therapy, CSCs display several properties: higher degree of drug efflux activity, active DNA repair, high ROS levels, the tendency of VM; moreover, non-CSCs may reacquire CSC properties by EMT, microenvironment, autophagy and extracellular vesicles also contribute to tumor relapse

Accumulating evidence shows that the CSCs are critically associated with drug resistance: ionizing radiation induces the upregulation of CD133^+^ CSCs in glioblastoma xenografts^99^ and CSCs are enriched in breast cancer after radiation therapy.^100^ Moreover, increasing evidence has demonstrated that acquired resistance to one specific drug can result in cross-resistance to other chemotherapeutics.^101–103^ CSCs can resist therapy mainly because they express multidrug resistance (MDR) transporters and display a more active DNA repair capacity and induce more apoptotic arrest than other cells.^104^ Therefore, researches should focus on CSCs to reverse therapy resistance (Fig. 2).

#### Increased drug efflux activity of CSCs

ABC transporters can export wide range of toxic substrates from cells^105^ and thus directly contribute to the acquisition of resistance and CSCs exhibit increased ABC transporter expression.^106^ CSC-mediated drug resistance is supported by the hypothesis that the SP fraction can be identified as CSCs. The number of ABC transporters was shown to be correlated with maturation state: cells that exhibit the greatest efflux activity are the most primitive.^76,107^ ABCG2 was the first ABC transporter reported to determine the SP phenotype.^75^ A more comprehensive study was conducted in 2001 and the results indicated that ABCG2 was a determinant of the SP phenotype and could be a marker for SCs from various sources.^75^ Various factors such as glutamine,^108^ DNA methyltransferase activity^109^ and hypoxia-inducible factor (HIF)^110^ can regulate the SP population by controlling ABCG2. However, the role of ABCG2 in the SP fraction is controversial: the ABCG2^+^ population did not show significant drug resistance compared with the ABCG2^-^ population. Moreover, ABCG2^-^ cells exhibited higher sphere formation than ABCG2^+^ cells,^111^ which is consistent with the findings in a study published more than 10 years ago.^112^ Patrawala indicated that ABCG2^+^ cancer cells can generate ABCG2^-^ cells and ABCG2^-^ cancer cells can also generate ABCG2^+^ cells.^112^ Another study showed that ABCG2 activity was not responsible for the stem-like phenotypes of CSCs.^113^ At present, the more pertinent conclusion is that the SP fraction is composed of heterogeneous cell populations. ABCG2 expression mainly identifies fast-cycling tumor progenitors, and the ABCG2^-^ population contains primitive stem-like cancer cells in the SP fraction. Other subtypes of the ABC transporter family also contributed to CSC-mediated chemoresistance: ABCB5 was colabeled with CD133^114^ and CD44^115^ and clinically correlated with chemoresistance.^115^ Furthermore, ABCB5 controls chemoresistant and ABCB5 blockade-induced cellular differentiation,^116^ which is possibly mediated by a cell cycle checkpoint mechanism.^117^ ABCB1 is another important ABC transporter contributing to the chemoresistance-phenotype of CSCs^118^ by PKC/PI3K/Akt.^119^

Although specific ABC transporters are inhibited, cancer cells display an MDR phenotype. Tepotinib significantly reversed ABCB1-mediated MDR but not ABCC1-or ABCG2-mediated MDR.^120^ While this phenotype can protect cells from cytotoxic agents, MDR genes are sensitive to drugs such as doxorubicin,^121^ motixantrone.^122^ Currently, (i) the expression of multiple ABC transporters in CSCs can compensate the effect of the inhibition of a single ABC transporter, (ii) the key regulatory SP-related pathways that modulate ABC transporter expression are poorly understood, and (iii) limited research on and comprehension of the characteristic of the SP fraction are the main reasons for clinical failure.

#### Enhanced capability of DNA repair in CSCs

The hypothesis that therapy resistance is due to increased levels of ABC transporters cannot explain the enhanced therapy resistance of CSCs. Cancer cells show decreased DNA repair and thus display many mutations and genomic instability, ultimately resulting in apoptosis after multiple therapies. CSCs have highly active DNA repair mechanism, which results in effective DNA protection.^123–125^

In addition to chemotherapy drugs causing DNA damage, radiotherapy can induce DNA damage. In short, radiotherapy induced DNA damage, directly or indirectly, via the production of water-derived radicals and reactive oxygen species (ROS), which then interact with macromolecules, including DNA, lipids and proteins. Then, the DNA repair machinery is induced. Distinct from normal cancer cells, CSCs have both lower ROS levels and enhanced DNA damage repair.

CSCs have low levels of ROS due to increases in ROS scavengers to reduce ROS-induced DNA damage and apoptosis,^126,127^ and the ROS scavenger N-acetylcysteine (NAC) restored the CSC phenotypes.^128^ Salinomycin, a compound that can selectively eradicate CSCs, could target the CD44^+^CD24^-^ fraction and upregulate ROS levels.^129^ NRF2 silencing reversed the ability of CD44^+^ cells to retain high levels of ROS and the sensitivity to anticancer drugs.^130^ Depletion of glutamine decreased the proportion of SP cells by increasing the intracellular ROS levels;^131^ glycolysis promoted the expression of doublecortin-like kinase 1 (DCLK1) and maintained the CSC phenotypes via maintenance of low ROS levels in gemcitabine-resistant pancreatic cancer cells.^128^ However, Lee et al.^132^ revealed that myelocytomatosis oncogene (MYC) and myeloid cell leukemia-1 (MCL1) cooperate to maintain chemotherapy resistance of CSCs by increasing ROS production and HIF-1α expression, which might be explained by the independence of the apoptosis domain. Inhibition of HIF-1α blocked CSC expansion and restored the sensitivity to chemotherapy.

The key sensors of DNA damage are the ataxia telangiectasia mutated (ATM) and ataxia telangiectasia mutated-RAD3-related (ATR) protein kinases. Upon DNA damage, ATM and ATR kinases form complexes with poly ADP-ribose polymerase (PARP-1) and breast cancer 1 (BRCA1) to phosphorylate checkpoint kinase 1 (CHK1) and CHK2, which subsequently drive the activation of targeted proteins, inducing DNA repair. CHK-associated contributors lead to therapy resistance of CSCs: a clinical cohort indicated that CHK1 phosphorylated at serine 345 is a predictor of radioresistance in breast cancer.^133^ Wang demonstrated that the c-MYC-CHK1/CHK2 axis regulates the DNA damage-checkpoint response and CSC characteristics, resulting in radiotherapy resistance.^134^ Srivastava found that enhanced expression of DNA polymerase contributes to cisplatin resistance in ovarian CSCs.^135^ In contrast, pharmacological inhibition of the DNA damage checkpoints CHK1 and CHK2 sensitized CSCs to radiotherapy.^99^ Furthermore, CSCs can develop high drug resistance through regulating their cell cycle. During chemotherapy, the cell cycle of CSCs slows down and the cells fall into a “quiescent” state. In this state, protective mechanisms can be initiated by the DNA damage repair system. When the DNA damage repair finished, CSCs resume tumorigenesis and thereby escape apoptosis.

Despite the interest and investment of pharmaceutical companies in the development of treatments that prevent DNA repair in cancer cells, the results tend to be worse than expected. The novel targets proposed herein proposed provide inspiration.

#### Strong correlation od CSCs with metastasis

Metastasis is a complex cascade of events including tumor cell conversion into mobile tumor cells, invasion into blood vessels, survival in circulation, attachment to endothelial cells in vessels, extravasation and finally colonization and growth in the host organ. During epithelial mesenchymal transition (EMT), epithelial cancer cells lose their polarity and cell-cell contacts, generating a mesenchymal phenotype with migratory and invasive characteristics. Although EMT is present in most cancer cells and not specific to CSCs, mobile CSCs might derive from stationary CSCs through the acquisition of a transient EMT phenotype except stemness. Paget compared metastatic cancer cells to “seeds” that, once released from the plant (primary tumor), can spread, survive and proliferate when on “congenial soil.”^136^ Although the metastatic process is considered highly inefficient because only a cluster of cancer cells can drive metastasis following their transplantation into immunodeficient mice,^137^ which is consistent with the low percentage of CSCs in cancer, the self-renewal and differentiation of CSCs play an important role: genome sequencing showed metastatic clones are genetically evolved from the original cells.^138^

Therefore, acquiring metastatic characteristics is the first step that may be mediated by EMT in CSCs. A clinical analysis of β-catenin^+^ cancer cells (strong indication of stemness) that coexpressed E-cadherin and vimentin in core-needle biopsies from patients with various advanced metastatic carcinomas showed a significant association among CSCs, EMT and metastasis.^139^ Recent studies have shown that, several signaling pathways and molecules play an important role in this process. SOX8 bound to the promoter region of Frizzled 7 (FZD7) and mediated EMT processes in chemoresistant tongue squamous cell carcinoma (TSCC) via the FZD7/Wnt/β-catenin pathway.^140^ Moreover, extracellular matrix 1 (ECM1) regulated Wnt-mediated EMT by increasing the association between β-catenin and MUC1 cytoplasmic tail.^141^ Twist1 is a basic helix-loop-helix transcription factor that potently drives the EMT process. Metadherin (MTDH) indirectly activates Twist1 expression by facilitating histone H3 acetylation on the Twist1 promoter, a process mediated by the histone acetyltransferase cAMP response element-binding protein-binding protein (CBP), resulting in CSC traits and drug resistance.^142^ Moreover, S-phase protein kinase 2 (Skp2) regulates castration-resistant prostate cancer through Twist-mediated EMT and CSC acquisition. Skp2 interacts with Twist and promotes the nondegradative ubiquitination of Twist. Consequently, Skp2 stabilizes Twist protein expression by preventing proteasomal degradation of Twist by β-TrCP.^143^ Zinc-finger E-box-binding homeobox-2 (EZB2) is an EMT inducing transcription factor. The FBXW7-ZEB2 axis links EMT and the tumor microenvironment (TMV) to promote colorectal CSCs and chemoresistance.^144^

The EMT provides a basis for CSCs with unique tendencies, which allows them to be better leveraged for treatments that are more strategic than treatments employing non-CSCs. So we expect that a successful CSCs therapy might be achieved by preventing the induction of EMT, selectively killing CSCs during the EMT process or pharmacologically inducing the reversal process (EMT to MET).

After metastasizing and infiltrating into the surrounding parenchyma, tumor cells enter the blood circulation, where they can induce anoikis.^145^ CSCs can evade anoikis and enter the circulation to reach distant target organs. Grillet and colleagues^146^ reported that circulating tumor cells (CTCs) from patients with CRC displayed CSC hallmarks in ex vivo culture. Moreover, a transgenic mouse model demonstrated that CTCs returned to the primary tumor and generated new tumors with enhanced tumorigenic capacity.^147^ Mechanistic investigations demonstrated that overexpression of stromal-derived factor-1γ (SDF-1γ or CXCL12γ) induced CSC phenotypes in prostate cancer cells through CXCR4-mediated PKCα/NFκB signaling^148^ and Wnt signaling,^147^ which promoted tumor outgrowth, metastasis and chemoresistance in vivo. For invasion of anoikis, androgen receptor (AR) may be important in CSCs: AR maintains a CSC-like tumor-initiating population and serves as an antiapoptotic factor, facilitating anchorage independence and metastasis^149^ and constant ubiquitination and degradation of AR by MDM2 conserves the CSC integrity.^150^

Finally, a single CSC could not survive alone after anoikis; other CSCs need to be preserved in the niche. A key factor that modulates the microenvironment and CSCs resulting in drug resistance is hypoxia.^151–153^ Hypoxia activates multiple signaling pathways by activating hypoxia-inducible factor-1α and 2α (HIF1α, HIF2α) or phosphatidylinositol 3-kinase (PI3K/AKT), which bind to promoters containing the hypoxia-response element (HRE) and then regulate gene expression. As a feedback loop, activation of the PI3K/ATK pathway promotes CSCs by activating HIF1α and HIF2α.^154^ The cascade of activation leads to the induction of stemness and self-renewal, which results in secondary tumors.

The development of new drugs targeting EMT program could have a significant impact on the CSC therapy field. However, effective targeting of CSCs still faces a variety of challenges, as the mechanism that regulates the retention or induction of EMT programs in CSCs remains unclear. Overcoming these challenges will require that the risk of resistance be minimized, but a successful therapeutic strategy will eventually open the door for curing cancer by targeting CSCs.

#### Vasculogenic mimicry (VM) in CSCs

VM is a newly defined pattern of tumor microvascularization that is different from angiogenesis and vasculogenesis and lacks the participation of endothelial cells, by which highly aggressive tumor cells can form vessel-like structures due to their high plasticity. VM channels provide a functional blood supply in malignant tumors and mediate therapy resistance. Increasing studies have found that CSCs directly line VM channels and provide VM-related molecules to enhance VM formation.^155^ Rates of CD133, ALDH, and VM were positively associated with lymph node metastasis, distant metastasis, Enneking stages, and overall survival of patients.^156^ A similar phenomenon was found: VM formation was associated with altered CSC-associated proteins,^157^ and CSCs directly line VM channels. Additionally, CSCs provide VM-related molecules to promote VM formation.^155^ CSCs can form VM-mediated resistance and acquire resistance to antiangiogenic therapy.^158,159^

#### Increased autophagic activity in CSCs

Autophagy is an evolutionarily conserved physiological process in cells that generates intracellular nutrients, growth factors and energy to support cell survival and cellular activities during stress, such as nutrition deprivation, hypoxia or ischemia.^160,161^ Autophagy was upregulated in CD133^+^ cells, and promoted resistance to photodynamic therapy (PDT).^162^ Moreover, CD44^+^CD117^+^ ovarian CSCs presented higher basal autophagy than their nonstem cell counterparts. Inhibiting autophagy could reduce chemoresistance in CSCs.^163^ The differential regulation of autophagy is a molecular link between the differing chemosensitivity of CSCs and differentiated cancer cells,^164^ consistent with the fact that ATG7 or ATG12 KD could decrease the pluripotency and promote the differentiation and/or senescence of CSCs.^165^ Some studies have revealed the mechanism by which autophagy facilitates the degradation of Sox2.^166^ Moreover, mitophagy could regulate the binding of the Nanog promoter to PINK1 via p53.^167^ Furthermore, ATG7 facilitated the transcription of Oct4 via β-catenin, which binds to the Oct4 promoter.^168^ TARBP2 was reported to be destabilized through autophagic-lysosomal proteolysis, thereby stabilizing the expression of Nanog.^69^

Overall, autophagy is an important mechanism activated by CSCs to increase their resistance to therapy. Autophagic inhibitors might decrease the stemness properties and reverse therapy resistance.

#### Decreased ferroptosis in CSCs

Ferroptosis is a recently described form of cell death that is distinct from other known cell death pathways.^169^ However, the precise mechanism of ferroptotic cell death is still unclear. Iron, ROS and lipid peroxidation are critical mediators of ferroptosis.^170,171^ The ability of iron to cycle between oxidized and reduced forms contributes to the formation of free radicals, and an excess of free radicals leads to lipid peroxidation, increased ROS and oxidative stress, and DNA damage.

CSCs are generally characterized by a high intracellular iron content.^172^ Iron addiction could be a therapeutic target in CSCs and could reverse therapy resistance.^173^ A forced reduction in intracellular iron reduced the proliferation of CSCs in OC. Moreover, CD44 expression suppressed ferroptosis in cancer cells, which indicated a correlation between CSCs and ferroptosis.^174^ Inhibition of autophagy increased the susceptibility of glioblastoma SCs to temozolomide by initiating ferroptosis.^175^ Furthermore, inducing ferroptosis could sensitize CSCs to chemotherapy in OC.^176^ In addition, as we mentioned before, salinomycin is a selective agent against CSCs and triggers ferritin degradation and ROS-mediated ferroptosis in CSCs,^177^ which may reverse the radiotherapy resistance caused by low ROS levels and enhanced DNA repair in CSCs.

However, few studies directly focusing on ferroptosis and CSC-mediated therapy resistance are available. Based on the association among ferroptosis and CSCs, CSC renewal and therapy resistance, further research should investigate the regulatory mechanisms of Oct4, Nanog and Sox2 by iron, ROS and lipid peroxidation, at both the transcriptional and translational levels.

#### Favorable TMV in the CSC niche

Accumulating evidence suggests that the TMV plays a crucial role in CSC development and is a potential target for therapy resistance. Extracellular vesicles (EVs), carcinoma-associated fibroblasts (CAFs), tumor-associated macrophages (TAMs) and chemokines in the CSC niche have important roles. Chemotherapy-induced EVs promote CSC traits and therapy resistance.^178^ The presence of Cav-1 in EVs acts as a potent driver to induce CSC phenotypes and can induce radio- and chemoresistance in recipient cells.^179^ Unfortunately, exosomes derived from gemcitabine-resistant pancreatic CSCs mediate the horizontal transfer of drug-resistant traits to gemcitabine-sensitive pancreatic cancer cells.^180^ Lysine-specific demethylase 1 (LSD1) expression was increased in CAFs as an upstream driver of Notch3-mediated CSC self-renewal.^181^ ZEB2-mediated induction of EMT was associated with stromal factors secreted from CAFs, which induced chemotherapy resistance.^144^ Furthermore, a specific subset of CAFs, CD10^+^GPR77^+^ CAFs promotes tumor formation and chemoresistance by providing a niche for survival of CSCs. Mechanistically, CD10^+^GPR77^+^ CAFs are driven by persistent NF-kB activation via p65 phosphorylation and acetylation.^182^ Although studies on TAMs are limited, Masahisa reported that TAMs produce milk fat globule epidermal growth factor 8 (MFG-E8), and MFG-E8 mainly activates Shh and further amplifies its anticancer drug resistance.^183^ Interestingly, the extracellular matrix (ECM) is also an integral part of the CSC niche that mediates therapy resistance. Hyaluronic acid in the ECM is a ligand for the CD44 receptor and can affect CSC stemness along with the response to differentiation therapy.^184^ Another component of the ECM, laminin-332, could reduce cell mitosis, present resistance to doxorubicin and sorafenib treatment, and increase the SP fraction.^185^

Therefore, crosstalk occurring in the TMV can expedite and confer resistance of CSCs to radiotherapy and chemotherapy. Elucidation of the content of the CSC niche would provide us with valuable information to design therapeutic targets, e.g., exosome-like nanovesicles could be used to specifically target CSCs.

#### Immune escape in CSCs

Immunotherapy has recently attracted global attention and has emerged as the “new hope” for cancer treatment. However, CSCs have developed many strategies to circumvent immune attack and maintain the immune-resistant phenotype.

PD-L1, a T-cell inhibitor expressed on T cells, B cells, and natural killer cells, can eliminate tumor cells.^186,187^ PD-L1 expression was substantially increased in chemoresistant CRC through the PI3K/Akt and MEK/ERK pathways;^188^ conversely, CSCs showed a low-immunogenic profile: ABCB5^+^ melanoma cells did not express the immunogenic differentiation antigen MART-1 at significant levels,^189^ nor did they express cancer testis antigens.^190^ Moreover, loss of the tumor suppressor PTEN in CSCs led to reduced expression of neoantigens that demonstrate strong immune reactivity and was thus associated with resistance to anti-PD-1 checkpoint blockade therapy.^191^ Furthermore, STAT3 signaling can functionally render CSCs immunosuppressive as inhibition of STAT3 can restore T-cell function.^192^ In addition, CSCs isolated from various solid tumors have been shown to release various cytokines and soluble immunosuppressive factors such as IL-4, IL-6, IL-10, and IL-13.^193^

### Signaling pathways of therapy resistance in CSCs

Various signaling pathways are involved in therapy resistance of CSCs. Some of the most important and well- characterized signaling pathways include Hedgehog(Shh), Wnt/β-catenin, Notch and NF-κB pathways.

#### Sonic Hedgehog pathway

**Fig. 3:**
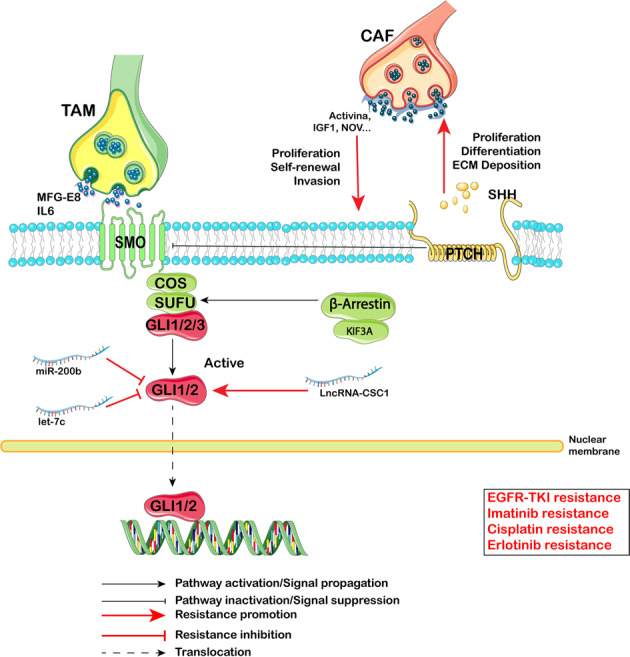
Hedgehog (Hh) signaling pathway-mediated therapy resistance in CSCs. Binding of the Hh ligand to its receptor Patched (PTCH) enables Smoothened (SMO)-mediated translocation of glioma-associated protein 1/2(Gli1/2) to the cell nucleus to drive the transcription of Shh target genes. During activation of the Hh pathway, some proteins (IL-6, MFG-E8), microRNA (miR-200b, let-7c) and the long noncoding RNA LncRNA-CSC1 are involved in the Hedgehog pathway to regulate EGFR-TKI resistance, Imatinib resistance, Cisplatin resistance, and Erlotinib resistance

The Sonic Hedgehog (Shh) pathway was initially identified in the fruit fly and has an important role in embryonic development. Binding of the Hh ligand to its receptor Patched (PTCH) enables Smoothened (SMO)-mediated translocation of glioma-associated protein 1/2(Gli1/2) to the cell nucleus to drive the transcription of Shh target genes^194^ (Fig. 3). Shh regulates the proliferation, differentiation and migration of target cells in a spatial, temporal and concentration- dependent manner.^195^ Enhanced Hedgehog activation can increase proliferation-associated genes: cyclin D1, cyclin D2, N-Myc, Hes1 and Igf-2.^195^ Shh is related to chemoresistance.^9^ Shh signaling regulates the ABCG2 efflux pump^196^ along with ALDH activity^144,197^ and reverses epidermal growth factor receptor tyrosine kinase inhibitor (EGFR-TKI) resistance. Furthermore, simultaneously inhibiting the Shh pathway could kill imatinib-sensitive or -resistant BCR^-^ABL^+^ cells.^198^ Moreover, Shh signaling maintained CSC phenotypes and malignant transformation phenotypes in CD44^+^ gastric cancer cells, and Shh inhibition could reverse chemotherapy resistance in CD44^+^ cells.^199^ LncRNA-cCSC1 activates the Shh signaling pathway and regulates the expression of CD44 and CD133.^200^ In contrast, miR-200b and let-7c significantly diminished Shh-mediated-erlotinib resistance in CSCs.^201^

From the perspective of the TMV, TAMs and CAFs contribute to Shh-mediated therapy resistance: TAMs produce MFG-E8, and MFG-E8 mainly activates Shh and further amplifies its anticancer drug resistance.^183^ Moreover, Shh participates in an intracellular signaling module that synergistically regulates CAFs and CSCs to mediate therapy resisitance.^202^

#### Wnt/β-catenin pathway

**Fig. 4:**
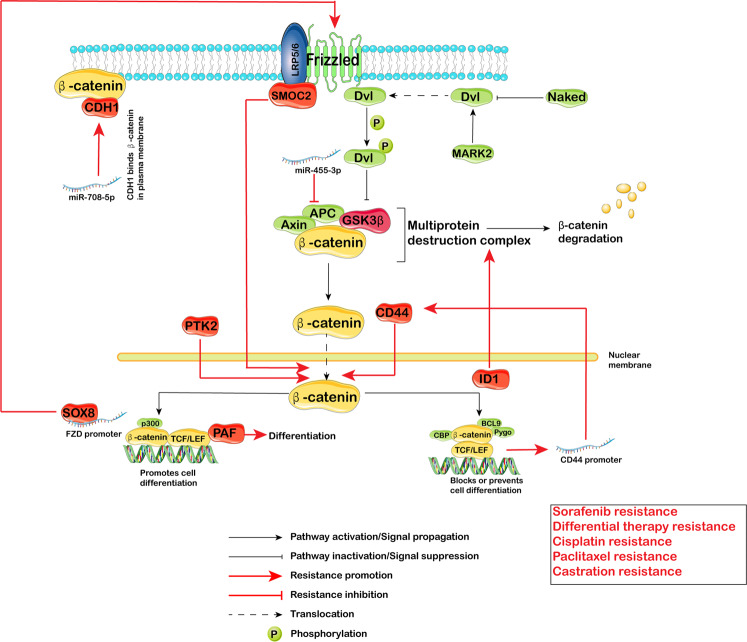
Wnt/β-Catenin signaling pathway-mediated therapy resistance in CSCs. In the absence of Wnt signaling, β-catenin is bound to the Axin complex, which contains APC and GSK3β and is, phosphorylated, leading to ubiquitination and proteasomal degradation through the β-Trcp pathway. In the presence of Wnt signaling, the binding of LRP5/6 and FZD inhibits the activity of the Axin complex and the phosphorylation of β-catenin, allowing β-catenin to enter the nucleus, and then bind to TEF/TCF to form a complex, which then recruits cofactors to initiate downstream gene expression. Several proteins (CDH1, SMOC2, SOX8, PAF, PTK2, CD44 and ID1) along with miR-708-5p regulate Sorafenib resistance, Differential therapy resistance, Cisplatin resistance, Paclitaxel resistance and Castration resistance

Wnt/β-catenin signaling plays a crucial role during embryogenesis. In general, the Wnt signaling pathway can be divided into canonical Wnt signaling (through the FZD-LRP5/6 receptor complex, leading to depression of β-catenin) and noncanonical Wnt signaling. Canonical Wnt signaling is the best-known (Fig. 4). In the absence of Wnt signaling, β-catenin is bound to the Axin complex, which contains APC and GSK3β and is, phosphorylated, leading to ubiquitination and proteasomal degradation through the β-Trcp pathway. In the presence of Wnt signaling, the binding of LRP5/6 and FZD inhibits the activity of the Axin complex and the phosphorylation of β-catenin, allowing β-catenin to enter the nucleus, and then bind to TEF/TCF to form a complex, which then recruits cofactors to initiate downstream gene expression.

The Wnt/β-catenin pathway regulates CSC-mediated therapy resistance: PTK2 promoter hypomethylation induces PTK2 overexpression and activates Wnt signaling, leading to the CSC phenotype and sorafenib resistance in HCC.^203^ MiR-708-5p could inhibit CSCs by repressing the Wnt pathway through promotion of CDH1 to bind β-catenin in the plasma membrane, resulting in loss of the release of β-catenin.^204^ PAF could induce the differentiation and lose of stemness of CSCs by binding β-catenin in a chemoresistance model.^205^ A more direct association between differentiation and therapy resistance was identified by Xiong Jin: ID1, which is important in lineage differentiation, could sensitize glioma CSCs to differentiation therapy by inhibiting β-catenin degradation,^206^ similar to the function of miR-455-3p.^207^ Moreover, SMOC-2 could activate Wnt by binding FZD6 and LRP6, resulting in paclitaxel resistance and cisplatin resistance.^208^ Furthermore, Wnt signaling is a key pathway regulating the well-known SC marker CD44 by directly interacting with the promoter and presenting castration resistance.^209^ Combined with the research conducted by Souvick Roy,^210^ these findings indicate a positive feedback mechanism between CD44 and Wnt: CD44 binds to β-catenin and activates Wnt, resulting in cispatin resistance. In addition, the transcription factor Sox8 was reported to promote the Wnt/β-catenin pathway by binding to the promoter of FZD7, eventually leading to cisplatin resistance.^140^

#### Notch pathway

**Fig. 5:**
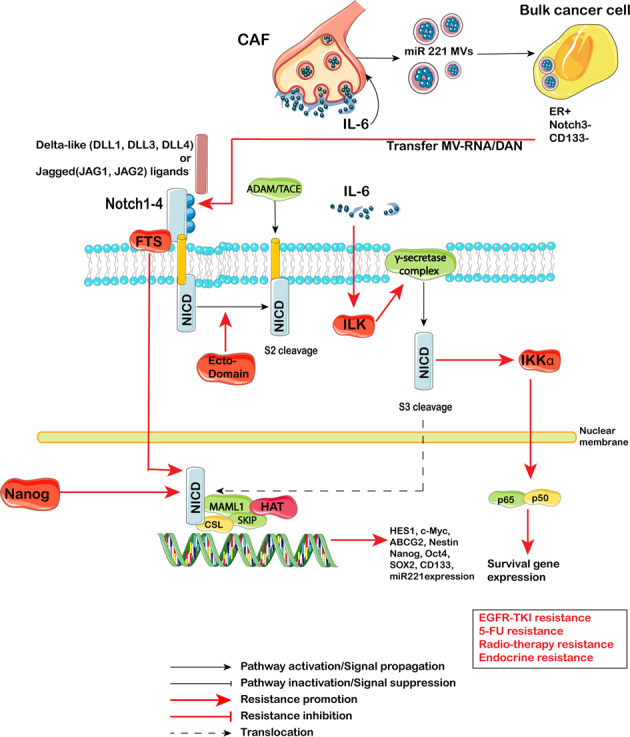
Notch signaling pathway-mediated therapy resistance in CSCs. DLL1, DLL3 and DLL4, and Jagged ligands (JAG1 and JAG2) expressed on the cell surface can induce signaling in adjacent cells expressing their cognate receptors Notch1–4. Ligand binding promotes sequential cleavage of the Notch receptors by ADAM/TACE enzymes (S2 cleavage) and then γ-secretase (S3 cleavage), resulting in release the NICD, which interacts with transcriptional regulators in the nucleus to induce a Notch gene-expression profile. Some proteins (FTS, ILK, Nanog) along with CAF and EVs regulate EGFR-TKI resistance, 5-FU resistance, Radio-therapy resistance and Endocrine resistance

DLL1, DLL3 and DLL4, and Jagged ligands (JAG1 and JAG2) expressed on the cell surface can induce signaling in adjacent cells expressing their cognate receptors Notch1–4. Ligand binding promotes sequential cleavage of the Notch receptors by ADAM/TACE enzymes (S2 cleavage) and then γ-secretase (S3 cleavage), resulting in release the NICD, which interacts with transcriptional regulators in the nucleus to induce a Notch gene-expression profile (Fig. 5). Notch target genes, in turn, regulate pivotal cell-fate choices, including differentiation, cell cycle progression, and survival.^211^

CSCs activate the Notch pathway to promote of resistance to chemotherapy^212^ and radiation.^213^ Inhibiting Notch signaling could sensitize CSCs to cisplatin or carboplatin^214^ and radiation therapy.^213^ Nanog regulated Notch signaling along with ALDH activity and radiotherapy resistance in breast cancer.^68^ Moreover, crosstalk between Notch and NF-κB contributed to therapy resistance in triple-negative breast cancer (TNBC):^215^ Jagged1 triggers nuclear, NF-κB-dependent transcription of antiapoptotic gene cIAP-2. Furthermore, extracellular signals can regulate Notch. En-Chi Hsu reported the indispensable role of ILK in regulating IL-6-induced Notch1 activation and CSC expansion through γ-secretase assembly at the caveolae.^216^ In addition, inhibiting ADAM-17, a major component of Notch signaling, by Nectin-4, could partially reserve 5-FU resistance.^217^ FTS could bind with Notch1 and then activate Notch signaling and upregulate Nanog, Oct4 and Sox2 expression, which contributed to radiotherapy resistance.^218^ TMV also participated in regulation of the CSC phenotype regulation. Stroma microvesicles mediated CSC evolution in endocrine resistant metastatic breast cancer.^219^ Autocrine IL-6/Stat3 signaling induces the proliferation of CAFs and the biogenesis of onco-miR221/222^+^ MVs; these MVs are taken up by estrogen receptor^+^ (ER^+^) breast cancer cells and lead to the potent suppression of ER signaling, resulting in Notch3 upregulation, which in turn sustains the self-renewal of CD133^+^ CSCs in an ER-independent manner.

#### NF-κB pathway

**Fig. 6:**
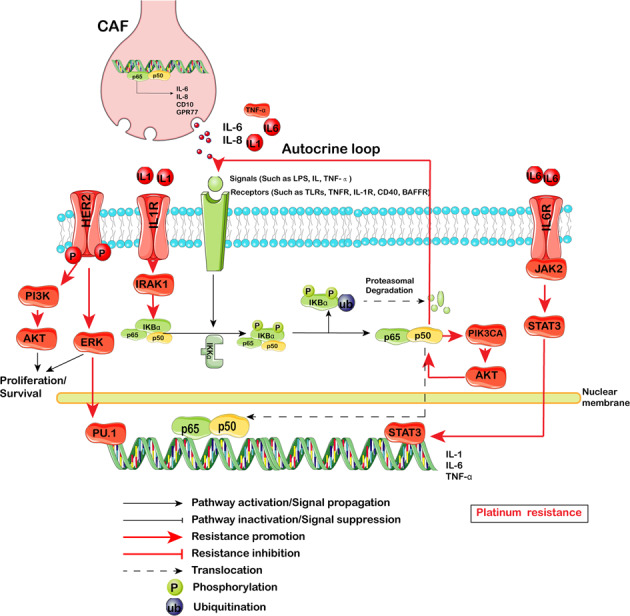
NF-κB signaling pathway-mediated therapy resistance in CSCs. The main physiological component of NF-κB is the p50-p65 dimer. The active p50-p65 dimer is further activated by post-translational modification and transported into the nucleus, inducing the expression of target genes in combination with other transcription factors. PI3K/Akt, ERK, IRAK1, Jak2/STAT3 and CAF regulate Platinum resistance

The NF-κB pathway mediates acute and chronic inflammation in tumors through the association of inflammation with stemness;^220^ it plays a crucial role in tumor biology and regulates key processes during the initiation and progression of various carcinomas.^221,222^ The main physiological component of NF-κB is the p50-p65 dimer. The active p50-p65 dimer is further activated by post-translational modification and transported into the nucleus, inducing the expression of target genes in combination with other transcription factors (Fig. 6).

More recently, NF-κB signaling was found to be preferentially activated in CSCs.^223,224^ Salinomycin, an inhibitor of NF-κB, could induce apoptosis in cisplatin-resistant OC.^225^ Furthermore, NF-κB participates in the progression of EMT: Twist2 transcriptionally enhances NF-κB activation, and NF-κB upregulates Twist2 expression, thereby forming a positive feedback loop that activates EMT and enhances CSC-like properties.^226^ Moreover, NF-κB activated hypoxia related stemness signaling,^227^ and revertes ROS-induced apoptotic cell death in CSCs. In addition, a model of HER2-induced sequential activation of the IL-1α and IL-6 signaling pathways was supported by the following evidence: (i) HER2 upregulates IL-1α expression via MAPK-mediated activation of the PU.1 transcription factor; (ii) secreted IL-1α binds to its receptor and activates NF-κB, which subsequently binds to and activates the *IL1A* and *IL6* promoters via a feedback mechanism; (iii) secreted IL-6 binds to its receptor and activates the downstream STAT3 transcription factor.^228^ The HER2/NF-κB model could promote tumorigenesis and chemotherapy resistance. Another autocrine loop was reported by Bhushan Thakur: cisplatin mediated NF-κB activation only in CSCs, which in turn activated the bimodal feedback loop of NF-κB-TNFα and NF-κB-PIK3CA. On the one hand, this mechanism promotes an autocrine loop by activating TNFα-NF-κB in CSCs, and on the other hand, it increases PIK3CA and PI3K/AKT signaling thus leading to NF-κB stabilization. Activated PI3K/AKT confers resistance against cisplatin through modulation of antiapoptotic (increase in cFLIP) and proapoptotic (decrease in Bax and PUMA) genes. A constant supply of NF-κB through the TNFα-NF-κB autocrine loop and enhanced stabilization of NF-κB by activated AKT maintains an antiapoptotic, quiescent CSC state that confers survival against chemotherapeutics in resistant cells.^229^ Similar to other signaling pathways, complement signaling maintains NF-κB activation in the TMV. CD10^+^GPR77^+^ CAFs promote tumor formation and chemoresistance by providing a niche for CSC survival. Mechanistically, CD10^+^GPR77^+^CAFs are driven by persistent NF-κB activation via p65 phosphorylation and acetylation, which is maintained by complement signaling via GPR77, a C5a receptor.^182^

#### RhoA/ROCK pathway

RhoA is the founding member of the Rho GTPase family, which also includes Cdc42 and Rac1.^230^ RhoA acts through Rho-associated, coiled-coil-containing protein kinase (ROCK) to control processes such as actin-myosin-dependent cell contractility, cell motility, and the cell cycle. Currently, a few groups have unveiled the significant role of RhoA/ROCK in CSC therapy resistance.^231^ In diffuse-type gastric adenocarcinoma (DGA), RhoA signaling promotes CSC phenotypes, which mediate cisplatin resistance.^232^ RhoA is involved in upregulating MDR1 in CSCs thus promoting drug resistance in CRC.^233^ Ephrin-B2 signaling also promoted tumorigenesis in a cell-autonomous manner, by mediating anchorage-independent cytokinesis via RhoA in glioblastoma stem-like cells (GSCs).^234^ The cyclin-dependent kinase 7/9 (CDK7/9) inhibitor SNS-032 repressed the transcription of the RhoA gene, and thereby decreased RhoA GTPase activity and actin polymerization, reducing the frequency of CSCs.^235^

### Overcoming therapy resistance of CSCs by prospective agents: from experimental research to clinical evaluation

Although the ability to target these resistant cell populations is approaching fruition, the majority of currently available anti-CSC strategies target stemness-associated factors, which are shared between CSCs and normal SCs. The therapeutic window of these approaches remains unclear. A more comprehensive understanding of CSC-specific targets, optimization of dosing relative to biological function, and the use of rationally designed combination strategies based on data from relevant preclinical models will yield an improved therapeutic window and targeting efficacy. For the above signaling pathways, which may contribute to CSC-mediated therapy resistance, new strategies targeting CSCs and the results of anti-CSC clinical trials (Table 2) will be discussed in detail below. Several factors limit the interpretation of the results of these trials: (i): Most of these studies lack robust SC readouts to prove the efficacy of drugs that directly target CSCs. (ii): For ethical reasons, most clinical trials are conducted with combined treatment for efficiency and safety. Most of these studies were not designed to target only CSCs. Therefore, while providing a mechanistic view of anti-CSC therapeutics, we preferred to focus on trials that reported subanalyses showing that the actual CSC compartment was targeted. In addition, studies on the proficiency of protein kinase inhibitors (PKIs) have shown cutting-edge results in reversing therapy resistance. Multikinase inhibitors such as regorafenib, sorafenib and EGFR-TKIs are discussed as below.

**Table 2:** Emerging agents targeting CSC-associated pathways

Drug class/mechanism	Agent	Experimental research	Suggested patient population	Notes	Phase
Agents targeting the Sonic Hedgehog pathway					
SMO antagonists	Vismodegib (GDC-0449)	GDC-0449 could inhibit stemness209 and reverse erlotinib resistance, radiation and carboplatin resistance;^258^	Multiple basel-cell carcinomas (MIKIE)^239^	Good activity in long-term regimens of MIKIE	2
			TNBC^240^	Downregulates CSC markers expression and sensitizes tumors to docetaxel	1
			Myelofibrosis^241^	Not improved any of the efficacy outcome	1b
	Sonidegib (LDE225)	LDE225 could destroy CSCs niche and reverse docetaxel resistance.^240^	TNBC^242^	No drug-to-drug interactions between sonidegib and docetaxel were found in the PK assessment	1b
			mBCC^243^	Sonidegib continued to demonstrate long-term efficacy and safety in mBCC.	2
SMO inhibitors	Glasdegib (PF-04449913)		Myelofibrosis^244^	Further study of glasdegib in combination with JAKi in a MF population may be warranted	1b/2
	Taladegib (LY2940680)		Advanced solid tumors^245^	Taladegib doses of 100 mg and 200 mg were well tolerated in this population of Japanese patients with advanced solid tumors.	1
			BCC^246^	LY2940680 treatment resulted in an acceptable safety profile in patients with advanced/metastatic cancer	1
	Saridegib (IPI-926)		Advanced Pancreatic Adenocarcinoma^247^	The study closed early	1
Agents targeting Notch pathway					
γ-secretase inhibition (GSI)	MK-0752		Pancreatic cancer^257^	Tumor response evaluation was available in 19 of 33	1
	RO4929097	RO4929097 reverse antiandrogen resistance,^259^ radiation resistance,^260^ and tamoxifen resistance^261^ mediated by CSCs;	Recurrent Malignant Glioma^263^	Combination of antiangiogenic and notch signaling inhibitors should be considered	1
			Glioma^262^	A specific decrease in the CD133^+^ CSC population	0/1
	PF-03084014	PF-03084014 reverse docetaxel resistance in CSCs.^265^	Advanced TNBC^268^	16% of 25 response-evaluable patients achieved a confirmed partial response	1
			Desmoid Fibromatosis^269^	Objective response rate of 71.4%	1
			Aggressive Fibromatosis^270^	PF-03084014 was well tolerated and demonstrated promising clinical benefit in patients	1
DLL4 inhibitors	Demcizumab (OMP-21M18)		Metastatic Non-Squamous NSCLC^271^	50% had objective tumor responses	1b
Agents targeting Wnt/β-catenin pathway					
Ligand sequestration	OMP-54F28 (FZD8-Fc)		Advanced solid tumors^249^	Agent was well tolerated	1
			Recurrent platinum-sensitive ovarian cancer^250^	75.7% of overall response rate	1b
Inhibitors of β-catenin	PRI-724	PRI-724 could downregulate expression of SOX2, CD44^251^ and reverse cisplatin resistance in CSCs;^252^	Hepatitis C Virus-related Cirrhosis^255^	Liver injury may be a possible related serious adverse event	1
	CWP232291	CWP232291 could reverse castration resistance in CSCs.^256^	NCT03055286	Recommended Phase 2 dose	1b
Agents targeting NF-κB pathway					
Nuclear export protein exportin 1 inhibitor	Selinexor	Selinexor could reverse paclitaxel resistance mediated by CSCs.^273^	Triple-class refractory multiple myeloma	Approved by FDA^29^	

#### Agents targeting the Shh pathway

As we noted previously, SMO activates a cascade. Vismodegib (GDC-0449) and sonidegib (LDE225) are oral SMO antagonists that have been approved by FDA.^236,237^

Ahmad and colleagues^201^ showed that erlotinib resistance was mediated by CSCs, and inhibition of the Shh signaling pathway by GDC-0449 resulted in the attenuation of CSC markers, leading to sensitization of EMT cells to drug treatment.^201^ Moreover, GDC-0449 could decrease stemness and both radiation and carboplatin resistance.^238^ Furthermore, in vivo treatment with GDC-0449 disrupted the intracellular signaling model mediated by Shh and reduced CAF and CSC expansion.^202^

A phase 2 trial has been conducted in patients with multiple basal-cell carcinomas treated with vismodegib,^239^ and both intermittent dosing schedules of vismodegib (group A and group B) seemed to show good activity in long-term regimens in patients with multiple basal-cell carcinomas. In the phase 1 clinical trial EDALINE, 3 of 12 patients with metastatic triple-negative breast cancer (TNBC) derived clinical benefit from combination therapy with a SMO inhibitor and docetaxel chemotherapy, with one patient experiencing a complete response.^240^ However, in the MYLIE study, which assessed the safety and efficacy of combining ruxolitinib with vismodegib in ruxolitinib-naive patients with myelofibrosis, no new safety concerns were reported, but the addition of vismodegib to ruxolitinib was not shown to improve the efficacy of the treatment.^241^

Sonidegib (LDE225) is another potent and selective SMO inhibitor. In mouse models of TNBC, Hedgehog ligand produced by neoplastic cells reprogrammed CAFs to provide a supportive niche for the acquisition of a chemoresistant, CSC phenotype via FGF5 expression and the production of fibrillar collagen. Stromal treatment of patient-derived xenografts with SMO inhibitors downregulated CSC marker expression and sensitizes tumors to docetaxel, leading to substantially improved survival and reduced metastatic burden.^240^

A phase 1 clinical study was designed to explore the combination of sonidegib plus docetaxel (fixed dose at 75 mg/ml) in advanced TNBC patients:^242^ no drug-to-drug interactions between sonidegib and docetaxel were found, and the combination showed antitumor activity in three of 10 patients with measurable disease. The median time to progression for the overall study was 42.5 days. 30-month analysis of the randomized phase 2 BOLT study was conducted to assess the long-term efficacy and safety of sonidegib in patients with locally advanced and metastatic basal-cell carcinoma. A possitive outcome was exhibited:^243^ patients treated with 200 mg sonidegib, had objective response rates of 56.1% (central) and 71.2% (investigator) in locally advanced basal-cell carcinoma and 7.7% (central) and 23.1% (investigator) in metastatic basal-cell carcinima.

Other selective SMO inhibitors, glasdegib (PF-04449913),^244^ taladegib (LY2940680),^245,246^ and saridegib (IPI-926)^247^ have entered clinical trials to be tested in various tumors. Since the safety profile of glasdegib monotherapy was manageable in patients with primary/secondary MF, further study of glasdegib in combination with JAK inhibitors in an MF population may be warranted. For taladegib, phase 1 dose escalation studies were designed and a low dose was tolerated in patients. The clinical efficacy of this drug should be further investigated. A phase 1 study of FOLFIRINOX plus IPI-926 for advanced pancreatic adenocarcinoma was closed early when a separate phase 2 trial of IPI-926 plus gemcitabine indicated detrimental effects of this combination.

Overall, despite the impressive preclinical activity and sheer number of trials with Hh inhibitors, the clinical efficacy of these agents has been modest.

#### Agents targeting the Wnt/β-catenin pathway

OMP-54F28 is a fusion protein that combines the cysteine-rich domain of Fzd8 with the immunoglobulin Fc domain that competes with the native Fzd8 receptor for its ligands and antagonizes Wnt signaling.^248^ Twenty-six patients were treated in a phase 1 study of the anticancer SC agent OMP-54F28^249^ and another phase 1b dose escalation study of OMP54F28 in combination with paclitaxel and carboplatin in patients with recurrent platinum-sensitive ovarian cancer. Further investigation is limited because of bone toxicity.^250^

PRI-724 is an inhibitor of β-catenin. PRI-724 reduced drug resistance and CSC phenotypes in TNBC^251^ and downregulated Sox2 and CD44 expression.^252^ Moreover, the combination of PRI-724 with cisplatin synergistically suppressed cell growth.^252^ While preclinical models showed that this treatment can reverse therapy resistance by targeting CSCs,^253,254^ its function in cancer has not been clinically determined, and liver injury may be a possible serious adverse event.^255^

CWP232291 is a small molecule Wnt/β-catenin inhibitor that blocks the growth of castration-resistant prostate cancer by activating the endoplasmic reticulum stress pathway.^256^ CWP232291 (NCT03055286) was evaluated in a phase 1b study of 45 patients with AML to determine the recommended phase 2 dose (RP2D) of CWP232291 in combination with cytarabine (ara-C) administered to subjects with relapsed or refractory AML. Published articles are currently unavailable.

#### Agents targeting the Notch pathway

γ-Secretase inhibitors (GSIs), such as MK-0752 and RO4929097, and the use of antibodies against the Notch receptor or ligand are the major clinical approaches targeting Notch signaling. A multicenter, nonrandom Bayesian adaptive design study of MK-0752 was performed to determine the safety of combination treatment and the recommended phase 2 dose (RP2D):^257^ tumor response evaluation was available in 19 patients; 13 achieved stable disease and 1 patient achieved a confirmed partial response. MK-0752 plus docetaxel could decrease CD44^+^CD24^-^ and ALDH^+^ cell fractions.^258^

RO4929097, another GSI, could sensitize prostate cancer cells to antiandrogen therapy.^259^ Moreover, RO4929097 could reduce IDO1 expression in cervical CSCs and reduce the binding of NICD on the IDO1 promoter, as well as sensitize xenograft tumors to radiation treatment.^260^ Furthermore, RO4929097 could overcome acquired tamoxifen resistance in CSCs in human breast cancer.^261^ In a phase 0/1 trial, 21 patients with newly diagnosed glioblastoma or anaplastic astrocytoma received RO4929097 combined with temozolomide and radiotherapy,^262^ and a specific decrease in the CD133 CSC population was observed. Thirteen subjects were enrolled in a phase 1 study of RO4929097 with bevacizumab in patients with recurrent malignant glioma.^263^The median overall survival was 10.9 months with a median progression-free survival of 3.7 months.

Finally, PF-03084014 is also a GSI. PF-03084014 inhibited HCC growth via suppression of cancer stemness.^264^ Moreover, PF-03084014 was reported to enhance the antitumor effect of docetaxel in prostate cancer stem-like cells.^265^ In pancreatic ductal adenocarcinoma (PDA), a combination of PF-03084014 with gemcitabine reduced putative CSCs. Notably, in a highly aggressive orthotopic model, a PF-03084014 and gemcitabine combination was effective in inducing apoptosis, and inhibiting tumor cell proliferation and angiogenesis, resulting in the attenuation of primary tumor growth as well as controlling metastatic dissemination, compared to gemcitabine treatment.^266^ Furthermore, a synergistic effect of PF-03084014 with docetaxel through targeting of CSCs was observed in breast cancer.^267^ Studies of PF-03084014 on advanced TNBC,^268^ desmoid fibromatosis,^269^ and aggressive fibromatosis^270^ demonstrated efficacy in desmoid tumors in phase 1 studies.

Demcizumab (OMP-21M18) is a first-in-class humanized antiDLL4 antibody. Twenty of 40 evaluable patients (50%) had objective tumor responses in the phase 1b trial of anti-CSC therapy.^271^ Moreover, demcizumab in combination with paclitaxel has a manageable toxicity profile and showed activity in patients with heavily pretreated platinum-resistant ovarian cancer.^272^ However, a recent study in metastatic pancreatic cancer failed to demonstrate survival benefit when demcizumab was added to gemcitabine plus Abraxane (YOSEMITE study) (NCT02289898). Another phase 2 study investigating the addition of demcizumab to standard first-line treatment with carboplatin plus pemetrexed in lung cancer (DENALI study) is ongoing (NCT02259582).

#### Agents targeting the NF-κB pathway

Selinexor is an oral inhibitor of the nuclear export protein exportin 1, which inhibits NF-κB signaling. Selinexor compounds synergize with gemcitabine and nanoparticle albumin-bound (nab)-paclitaxel, leading to suppression of pancreatic ductal adenocarcinoma (PDAC) growth and CSC spheroid disintegration.^273^ Recently, the FDA granted accelerated approval to selinexor plus low-dose dexamethasone for triple-class refractory multiple myeloma,^29^ because selinexor-dexamethasone resulted in objective treatment responses in patients who displayed resistance to several proteasome inhibitors.^274^ As it is for relapsed patients, selinexor may play a role in reversing therapy resistance. Combined selinexor and gemcitabine could suppress CSC spheroids in a PDAC phase 1b trial,^273^ identifying selinexor as a promising agent targeting CSCs.

#### Effects of protein kinase inhibitors (PKIs)

Given that accumulating evidence has demonstrated that eventual treatment failure resultes from multiple defense mechanisms of CSCs, we should block the compensatory responses induced by mutual communication in these cells. Several multikinase inhibitors have been approved for patients who failed to respond to currently available chemotherapeutic agents.^275^ The potential efficacy of PKIs on CSCs has been investigated: multikinase inhibitors (such as regorafenib and sorafenib) and a group of EGFR-TKIs are being examined.

Regorafenib is an oral multikinase inhibitor that blocks the activity of protein kinases involved in angiogenesis, oncogenesis, metastasis, and tumor immunity.^276,277^ Limited studies have indicated that regorafenib can reverse drug resistance caused by CSCs: regorafenib treatment decreased the stemness phenotypes including tumor sphere formation and the SP fraction of HCT-116R and DLD-1R cells. The combination of regorafenib and 5-FU significantly suppressed the tumorigenesis and stemness markers of 5-FU resistant cells.^278^ In addition, regorafenib could decrease the expression of CSC markers in PDAC.^279^ Moreover, targeting the TMV with regorafenib altered the tumor cell-marrow-derived mesenchymal stem cells (MSCs) interaction, which in turn inhibited the growth and metastasis of colon cancer.^280^ Furthermore, in an AML model, leukemic SCs were sensitive to regorafenib treatment.^281^

Increasing studies have found that combinatorial administration could possibly reverse therapy resistance in CSCs. The poor efficacy of first‑generation EGFR‑TKIs for lung adenocarcinoma appears to be related to the increased expression of CSC markers.^282^ Overexpression of shisa3 inhibited CSC properties in lung adenocarcinoma cells and reversed resistance to gefitinib/osimertinib, which are EGFR-TKIs.^283^ The EGFR-TKI, brexpiprazole, combined with osimertinib, is a potential therapeutic strategy for glioblastoma by chemosensitizing glioma CSCs through the downregulation of survivin expression.^284^

Sorafenib is another oral multitargeted receptor tyrosine kinase inhibitor, and although it significantly prolonged progression-free survival,^285^ its long-term success is quite low due to the development of resistant cells^286^ and adverse drug reactions (ADRs).^287^ Efforts should also be made to explore other potent molecular targets that can improve the efficiency of sorafenib. Huang’s team reported that lysine-specific histone demethylase 1A (KDM1A) inhibitors dramatically suppressed the stem-like properties of sorafenib-resistant cells by regulating the Wnt signaling pathway.^288^ An inhibitor of cyclin-dependent kinase 1(CDK1), RO3306, combined with sorafenib could potently decrease tumor growth in patient-derived xenograft (PDX) models, and the combined administration synergistically downregulated CDK1/β-catenin signaling as well as the pluripotency proteins Oct4, Sox2, and Nanog.^289^ NF-κB signaling mediated sorafenib resistance,^290^ and cotreating cells with sorafenib and sulforaphane downregulated NF-κB and reversed sorafenib-induced NF-κB binding, which was associated with decreased clonogenicity, spheroid formation, ALDH activity and migratory capacity.^291^

### Perspectives

Overall, CSCs are key players in tumorigenesis and, through multiple and different mechanisms, contribute to the therapy-resistant phenotype. Innovative treatments for CSC sensitization should include the combination of drugs targeting ABC transporters, DNA damage repair, metastasis, autophagic inhibition, ferroptosis and the TMV disruption and immunotherapies. The level of resistance may reflect the sum of alterations of different molecular pathways (in which resistance-related proteins are deregulated). From this point of view, the therapeutic approach needs to be extremely effective and efficient in space (tumor volume) and time (effective in the first-line of treatment). As noted previously, the number of patients in current clinical trials remains limited, which is not conducive to carrying out pivotal phase 3 trials. In addition, preclinical models of therapy resistant are usually conducted in normal cancer cell lines rather than CSC models. This condition could lead to errors: although normal cancer cell lines were killed by preclinical therapy, the CSC subpopulation still remained, which induces cancer recurrence. In addition, the extent to which the current marker-designated populations are actual CSCs remains to be answered. Improvement of preclinical models of CSC should be further undertaken, and identification of surrogate markers or functional assays to monitor biological activity and treatment responses are needed. The landscape of agents targeting CSC self-renewal pathways or TKIs is expanding, and combined treatments to avoid off-target effects is one notable strategy. Finally, the reversal of therapy resistance of CSCs is not straightforward and require the following: (i) improved understanding of the mechanisms regulating CSC resistance to therapy; (ii) a combination of pharmacology and pharmacology for design and structural modification of drugs; (iii) both experimental and molecular modeling simulations of crystal structures, properties and formation of targeted proteins and agents; (iv) some new strategies, e.g., targeting drug-efflux pumps, targeting the CSC niche and the quiescent state and induction of CSC apoptosis and ferroptosis.
